# The person-centred care game: a reflective tool for learning person-centred care in higher education

**DOI:** 10.12688/mep.19367.2

**Published:** 2023-07-21

**Authors:** Catarina Wallengren, Caroline Feldthusen, Ida Björkman, Emma Forsgren, Annie Jonnergård, Irma Lindström Kjellberg, Mari Lundberg

**Affiliations:** 1 Institute of Health and Care Sciences, Sahlgrenska Academy, University of Gothenburg, Gothenburg, Sweden; 2 University of Gothenburg Centre for Person-Centred Care (GPCC), Sahlgrenska Academy, University of Gothenburg, Gothenburg, Sweden; 3 Division of Physiotherapy, Department of Health and Rehabilitation, Institute of Neuroscience and Physiology, Sahlgrenska Academy, University of Gothenburg, Gothenburg, Sweden; 4 Department of Health Promoting Science, Sophiahemmet University, Stockholm, Sweden

**Keywords:** Person-centred care, serious game, reflection, higher education

## Abstract

Person-centred care (PCC) is being implemented within many health care systems and educational institutions are important enablers of learning PCC. Teachers in higher education are responsible for helping students develop the ability to reflect. One approach is with serious games, which allow students to reflect on realistic situations and shape their skills with virtual patients. This paper describes the development of a serious game, the person-centred care game – (PCC game), which was designed to promote learning of PCC by reflection. We demonstrated how this PCC game could be used to induce PCC knowledge and skills by student reflection in an academic course on PCC.

## Introduction

### Person-centred care

The World Health Organization highlights the importance of implementing health care that starts with the patient ( World Health Organization, 2015). This model of care is defined with various terms in various frameworks. The European standard ( CEN/TC 450 2020) term is ‘person-centred care’ (PCC), defined as an approach where ‘patients become more involved in their own care and treatment’.

### Serious game

Various learning activities have been developed to implement PCC ( Britten *et al*., 2020). One of these activities is called the PCC game. Games that are used to change the players' thinking or skills are called ‘serious games’. The overall purpose of serious games is to promote learning in a pleasurable way through reflection ( Laamarti *et al*., 2014). Games have been used in higher education ( Sharifzadeh *et al*., 2020) to increase student academic commitment, retention, performance ( Pechenkina *et al*., 2017), thinking, emotions ( Vlachopoulos & Makri, 2017), as well as motivation and academic skills ( Subhash & Cudney, 2018; Zeybek & Saygı, 2023). Further, a systematic review has shown that games enhance students' individual and group-based reflective abilities ( Krishnamurthy *et al.*, 2022), indicating that serious games can support students reflection within higher education system.

### Learning through reflection

Reflection can be seen as a process where learning takes place through action ( Dewey (1922/2002). Dewey argues that reflection is a process that entails individuals actively, persistently, and carefully examining, questioning, considering, and evaluation of thoughts and ideas in order to arrive at conclusions that can offer more profound understanding, i.e., to have grasped the meaning of something. In this way, reflection guides future action and thinking ( Dewey, 1991). Therefore, games should include questions, challenges, and information, because these elements trigger reflection ( Mekler *et al*., 2018). Hatton & Smith (1995) described four overall levels of reflection that can contribute to the development of knowledge and skills. The first level is ‘descriptive writing’, where the person writes down the events that are in focus. The second level is ‘descriptive reflection’, where the person describes their personal opinions and motives for actions. The third level is ‘reflective dialogue’, where the person examines motives, alternative solutions, and possible consequences of actions. The fourth level is ‘critical reflection’, where the person examines thoughts from a broad perspective. This study briefly describes the development and design of a computer-based PCC game. We tested the game for use in higher education to promote student reflections and learning on PCC.

## Methods

### Development of the PCC game

In 2016, the PCC game was created in a collaboration with patients, students, professionals, game developers, and researchers (founders and developers ILK, CW) recruited from the Centre for Person-Centred Care, University of Gothenburg, the Network for Health and Medical Care, and the software company IUS Innovation AB. Students from the University of Gothenburg, at medicine and nursing participated both in the development of the game and testing the prototype and final version, by written comments. The purpose of the game was to promote the players’ abilities to reflect and learn about PCC. The theoretical framework of the PCC game is grounded in the work of the University of Gothenburg Centre for Person Centred Care (GPCC) which is built upon Ricoeur's ethics ( Ekman *et al.*, 2011). This ethic declares that every person has the right to use their capacity, express their will and decide for themselves what constitutes a good life. Further, the GPCC framework ( Britten *et al.*, 2020; Ekman *et al.*, 2011) suggests that the ethics may be practiced by the three cornerstones listening to patient narrative, partnership, and documentation of a health plan. The core component of PCC is mutual respect and the co-creation of care through a partnership between patients, their families and careers, and health care professionals ( Ekman *et al.*, 2011). The PCC game was designed primarily for professionals, but also for patients and relatives and you choose to enter the game either as a healthcare professional or a lay person. The healthcare professional entry can also be used to promote healthcare students reflection and learning about PCC in the context of higher education.

### Design of the PCC game

**Figure 1.**
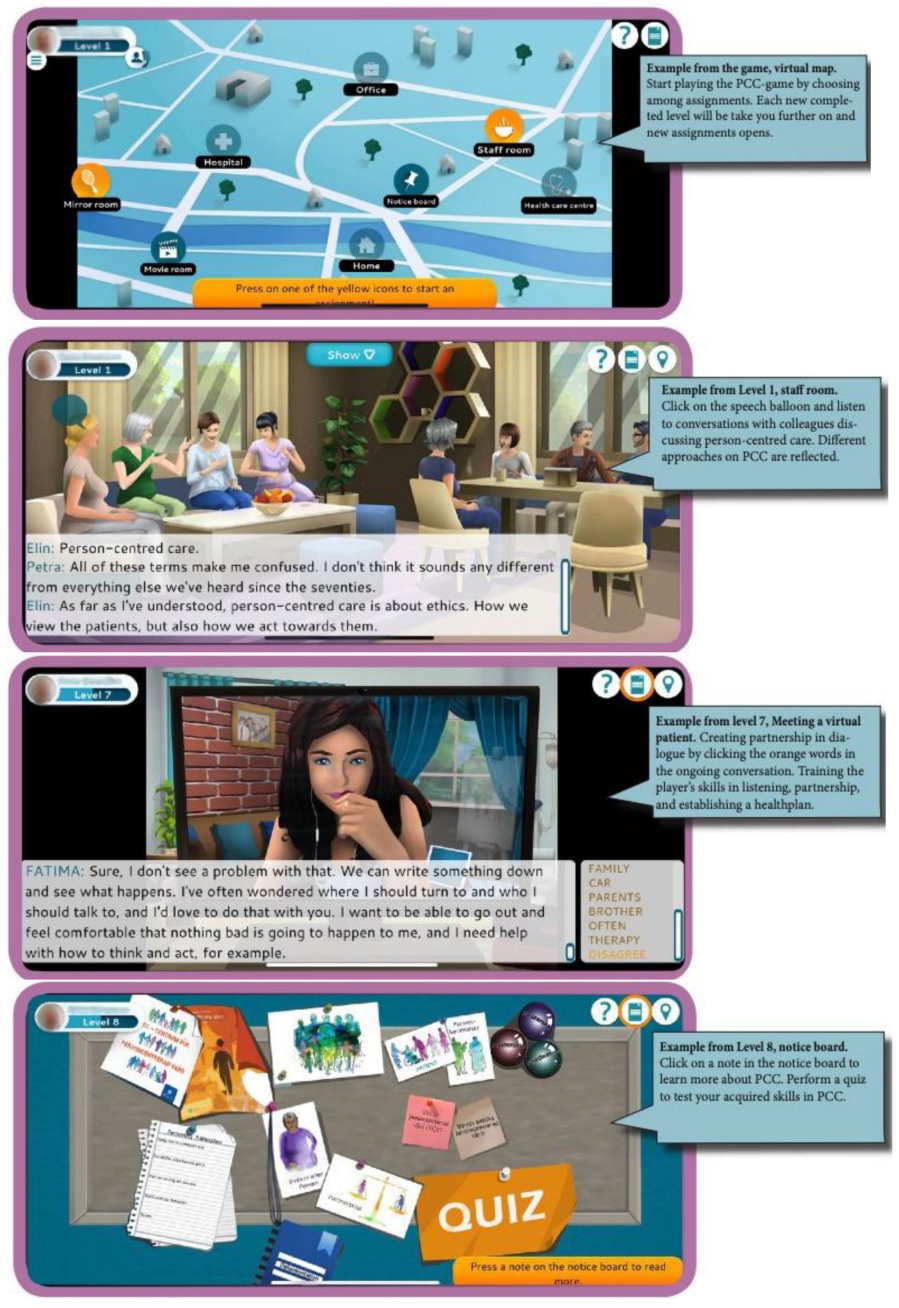
Selected pictures in the PCC game.

The PCC game is played on a mobile or tablet, and it consists of nine levels. It starts with the player creating a personal profile by answering following questions ‘Who am I?’ and ‘Why did I chose to work in health care?’ (Level 1). Thereafter, a virtual world opens up ( Figure 1), and the player enters a map, where the player can choose challenges within the game or in real world. When the player is within the game, he/she encounters virtual patients in a home environment (level 2), in a hospital context (level 3), in a primary care context (level 5), and in a digital meeting context (level 7). The meetings with virtual patients aim to train the player’s skills in listening, partnership, and establishing a health plan. The tasks encountered in the real world aim to train the player to develop listening and partnership skills in clinical activities (level 4), to identify what tasks can be person-centred at their clinical placement and/or workplace (level 6), and to establish health plans in clinical contexts (level 8). Level 9 tests the player’s knowledge of PCC with a quiz. In addition, players can watch, read, and listen to movies, literature, and myths about PCC.

During the development phase, the game was tested by patients, students, and professionals. The Swedish version of the PCC game was launched in 2017. ‘ PCV-spelet’ on App Store or Google Play. The English version was released in 2019. ‘ PCC game’ on App Store or Google Play.

## Use cases

### Use of the PCC game for learning PCC through reflection

We tested the PCC game in an interdisciplinary, higher education course including students in the process of becoming nurses, physicians, occupational therapists, physical therapists, pharmacists, dietitians, and speech therapists. All students had experiences from clinical practice either from clinical placement (internship) or employment, which was used in their reflections. One example for the second cycle course; Person-centredness in health and social care, is included as *Extended data* ( Wallengren *et al*., 2022). We conducted the course in five modules, which were accompanied by a standardized course guide also included as extended data. All students played all nine levels of the game over a period of five weeks ( Table 1). In each module, students were given tasks that increased the degree of reflection. Formative assignments and examinations regarding the students’ progression through the game were used.

**Table 1.** Academic teaching modules on how to implement person-centred care (PCC).

Course week (modules)	PCC game levels	Learning activities	Topics covered related to implementing PCC	Levels of reflection *
1	1 2 3	Questions Myths Virtual patients Dialogues Information	Narratives Human resources Partnership	Descriptive writing
2	4	Information New experience	Human Resources Health plans	Descriptive reflection
3	5 6	Questions New experience Dialogue	What routines are currently person-centred in clinical practice? How can we increase patient involvement in daily practice?	Internal reflective dialogue
4	7 8	Questions New experience Dialogue Establish alternative health plan	Health plan Partnership	Critical reflection
5	9	Questions	Partnership Person-centred care	Critical reflection

*according to Hatton & Smith (1995)

Module 1 aimed to initiate student reflection ( Table 1) with following questions; (1) What is your motive for starting work in health and social care? (Level 1); (2) what are your resources? (Level 2); and (3) what is the importance of forming partnerships with patients? (Level 3). To encourage students to reflect further, students could listen to recordings of a discussion on myths about PCC and two narratives given by virtual patients ( Table 1). The students' written descriptions of reflections were discussed with peers and teachers in a subsequent seminar.

Module 2 was designed to induce student reflection on medical practices by directing their attention to current patient health plans in health and social contexts ( Table 1). In this module (level 4), students were encouraged to think about why current routines were adequate or inadequate and to suggest alternative ways to develop patient documentation.

Module 3 focused on the student’s inner reflective dialogue, and it aimed to deepen their learning about PCC. Students were encouraged to study their own context and reflect on whether the care they currently provided was actually person-centred ( Table 1). Then, they were asked to reflect on how they might increase patient involvement in their clinical practices.

In module 4, students were encouraged to employ critical reflection. At this level, the student had to identify factors that facilitated or impeded the success of the health plan currently used in their practice. In light of these factors, they were instructed to develop an alternative health plan that engaged the patient as a partner in health and to consider how the plan could be implemented in clinical practice. Their proposals for new health plans would then be tested with actual patients (in the student’s work place). Furthermore, the PCC game encouraged students to give concrete suggestions about how their actions could become more person-centred ( Table 1).

In module 5, students continued to practice critical reflection by reflecting on how their ability to develop partnerships had evolved during their five weeks of participation in the course. Furthermore, they tested their level of knowledge with a quiz ( Table 1).

## Discussion/Conclusion

This study described the development and design of a serious game, which was created in partnership with several actors. The partnership ensured that the PCC game's learning tools (questions, films, myths) and content (resources, narratives, health plan) were authentic and consistent with everyday practices. Further, we have demonstrated how the PCC game could be used in connection with an academic course. Finally, we showed how the game could be used to deepen the student’s ability to reflect. This paper may be useful in arousing the curiosity of university professionals and enticing them to use the PCC game as a reflection tool for teaching PCC. However, the effects on learning PCC by playing the game needs to be further evaluated.

## Data avaliability

### Extended data

Zenodo: Extended data connected to 'The person-centred care game.' https://doi.org/10.5281/zenodo.7270786 ( Wallengren *et al*., 2022).

This project contains the following extended data:

- Local course syllabuses – Person-centeredness in health and social care, 7.5 higher education credits. Avancerad nivå / Second Cycle.
- Course guide – Guide for improved learning when using the person-centred care game.

Data are available under the terms of the Creative Commons Attribution 4.0 International license (CC-BY 4.0).
