# Supplementary material for: The person-centred care game: a reflective tool for learning person-centred care in higher education
Source: MedEdPublish (2016). 2023 Jul 21;13:2. Originally published 2023 Jan 9. [Version 2] doi: 10.12688/mep.19367.2 (PMC10423947; doi:10.12688/mep.19367.2)
Supplement: Peer review history

# Peer review: Reviewer response for version 2

**DOI:** 10.21956/mep.21144.r34213

**Published:** 2023-08-01

**Authors:** Margaretha Ekebergh

**Affiliations:** 1 Faculty of Caring Science, Work Life and Social Welfare, University of Borås, Borås, Sweden

I have reviewed the new version and I think this is appropriate. Especially the clarifying of the concept of reflection increase the status of the content. This definition is important to understand the purpose of the care game. From my perspective the article is now completed for indexing. I don’t have any more comments to the text.

Is the rationale for developing the new method (or application) clearly explained?

Yes

Is the description of the method technically sound?

Yes

Are the conclusions about the method and its performance adequately supported by the findings presented in the article?

Partly

If any results are presented, are all the source data underlying the results available to ensure full reproducibility?

Yes

Are sufficient details provided to allow replication of the method development and its use by others?

Partly

Reviewer Expertise:

Reflection and learning in caring contexts. Lifeworld oriented caring science with focus on the patient perspective.

I confirm that I have read this submission and believe that I have an appropriate level of expertise to confirm that it is of an acceptable scientific standard.

# Peer review: Reviewer response for version 2

**DOI:** 10.21956/mep.21144.r34212

**Published:** 2023-08-01

**Authors:** Victor Weng Keong Loh

**Affiliations:** 1 National University of Singapore, Singapore, Singapore

Thank you for clarifying that the focus of the game was to learn PCC through reflection, and that students playing the game would have had some experience of clinical practice and/or employment that would be the “matter” to reflect on.

 Congratulations and best wishes with the game.

Is the rationale for developing the new method (or application) clearly explained?

Yes

Is the description of the method technically sound?

Partly

Are the conclusions about the method and its performance adequately supported by the findings presented in the article?

No

If any results are presented, are all the source data underlying the results available to ensure full reproducibility?

No

Are sufficient details provided to allow replication of the method development and its use by others?

Partly

Reviewer Expertise:

Professional identity formation

I confirm that I have read this submission and believe that I have an appropriate level of expertise to confirm that it is of an acceptable scientific standard.

# Peer review: Reviewer response for version 2

**DOI:** 10.21956/mep.21144.r34211

**Published:** 2023-07-31

**Authors:** Clare Killingback

**Affiliations:** 1 Faculty of Health Sciences, Department for Sport, Health and Exercise Science, University of Hull, Hull, UK

Many thanks, I am satisfied that my comments have been addressed.

 I wish the authors all the best with their innovative reflective practice tool!

Is the rationale for developing the new method (or application) clearly explained?

Yes

Is the description of the method technically sound?

Yes

Are the conclusions about the method and its performance adequately supported by the findings presented in the article?

Partly

If any results are presented, are all the source data underlying the results available to ensure full reproducibility?

No source data required

Are sufficient details provided to allow replication of the method development and its use by others?

Yes

Reviewer Expertise:

Person-centred care, pedagogic research, physiotherapy

I confirm that I have read this submission and believe that I have an appropriate level of expertise to confirm that it is of an acceptable scientific standard.

# Peer review: Reviewer response for version 1

**DOI:** 10.21956/mep.20753.r33639

**Published:** 2023-06-15

**Authors:** Victor Weng Keong Loh

**Affiliations:** 1 National University of Singapore, Singapore, Singapore

Thank for you for the opportunity to review this manuscript

 Congrats to the team for developing the game which may potentially positively affect at least two important areas of health/social care practice - reflective practice, and person centredness (more specifically, person centred communication). Both important for attaining that golden fleece of health/social care practice - humanistic professional identity formation.

 However I am not clear what is the main focus of this game: whether the objective of the game is to strengthen reflective practice and skills which it seems to do (Level 1 to 9) or whether the objective is about strengthening PCC, the measurement or assessment of which, there is little mention in this manuscript. While there is a link between reflection and action (Dewey), there are jumps between reflection in a virtual context -> action taken in a virtual context -->> (bigger jump) reflection/action or practice in a real world context. On the other hand, the virtual space provides a safe space for rapid iteration and practice. The downside is it provides at best a high-fidelity surrogate of the real-world.

 In my experience, and assuming person centred care through communication is the outcome, curriculum/game designers need to give learners increasing doses of reality to learn/practice communicating in a person centred manner. A schema of progression might be - 

 Paper play -> Virtual play -> Simulation -> Real world context (simple) -> Real world (complex).

 So while the virtual context is safe and one can be creative about situations of/for learning, it remains a preliminary, and usually an early step in the train of learning/practice before being entrusted as a professional. The caveat being that simulations allow for the curation and creation of unusual/rare situations useful for learning, particularly useful when the opportunistic context is a rarity eg. rare conditions.

 This paper does well in giving a broad description of the theoretical rationale for a game to strengthen reflection and interestingly in a virtual world, which kudos to the team, has been developed with contribution from professionals, patients and family members.

 What would strengthen the manuscript would be to show the effects of playing the game on a specific group of learners. I imagine using the virtual context may be more effective for early learners eg. "preclinical students" rather than mature clinical students who have actual interactions with patients, family members, and care teams. And that for the more senior learner who has the opportunity to, reflection on actual experiences may be more directly effective in being critical about their/our (unconscious, reflex) behaviours in the actual work and practice. 

 To strengthen this paper, I suggest having a pilot with a designated group of learners and determining the outcome of playing this game. Questions which I am not sure of:

1. What type of learner (stage of experience) would benefit from playing this game;
2. Would a different learning methodology be more construct consistent eg. person centred care possibly better learnt through interactions (speech, emotions conveyed) with persons; virtual communications through text type communicating is quite different from the language spoken in the ward/clinic;
3. Have a think what outcome measure we want and how how best to measure it - the team needs to decide if it is about being more reflective? Being more person centered (in communications I presume); and if communications, is it with team members, patients, family members? Or is it about being more reflective about being person centred? Good to be crystal clear about what we intend to achieve here;
4. Outcome measure: perhaps self-rating tools (reflection or PCC as the research team decides) or qualitative methods.

In conclusion, my sense is that this serious game shows promise possibly for the early learner; and while I am less convinced of its value for the more senior care professional, this is merely my opinion. The answer will reveal itself in a carefully planned pilot with clear outcome goals/measures so that the effects of the PCC game in the serious work of person centred care will be made clearer for health professional educators.

 Best wishes to the team.

Is the rationale for developing the new method (or application) clearly explained?

Yes

Is the description of the method technically sound?

Partly

Are the conclusions about the method and its performance adequately supported by the findings presented in the article?

No

If any results are presented, are all the source data underlying the results available to ensure full reproducibility?

No

Are sufficient details provided to allow replication of the method development and its use by others?

Partly

Reviewer Expertise:

Professional identity formation

I confirm that I have read this submission and believe that I have an appropriate level of expertise to confirm that it is of an acceptable scientific standard, however I have significant reservations, as outlined above.

# Response

**Published:** 2023-07-11

**Authors:** Caroline Feldthusen

**Affiliations:** University of Gothenburg, Sweden

*The authors would like to thank the reviewer for valuable comments and suggestions which will enhance the manuscript and make the article clearer.*  Point by point respond:   

 However I am not clear what is the main focus of this game: whether the objective of the game is to strengthen reflective practice and skills which it seems to do (Level 1 to 9) or whether the objective is about strengthening PCC, the measurement or assessment of which, there is little mention in this manuscript.

*Respond: We agree, this is not clear. The focus is on learning PCC through reflection. We have revised the abstract to clarify this.* *In the abstract we have added:* *- “Person-centred care (PCC) is being implemented within many health care systems and educational institutions are important enablers of learning PCC.”* *- the PCC game was designed to promote “learning of PCC by reflection”,* *- and finally, that the PCC game could be used to induce “PCC knowledge and skills by” student reflection in an academic course on PCC.* *We hope this will make the focus of the article clearer.*  

 While there is a link between reflection and action (Dewey), there are jumps between reflection in a virtual context -> action taken in a virtual context -->> (bigger jump) reflection/action or practice in a real world context. On the other hand, the virtual space provides a safe space for rapid iteration and practice. The downside is it provides at best a high-fidelity surrogate of the real-world.

*Respond: The PCC game was developed for professionals, patients, and relatives to promote learning and reflection about PCC. However, we have also used it in health care students in a second cycle course to promote reflection which is now more clearly described in this method paper.* *Added in the manuscript:* *“The PCC game was designed primarily for professionals, but also for patients and relatives and you choose to enter the game either as a healthcare professional or a lay person. The healthcare professional entry can also be used to promote healthcare students reflection and learning about PCC in the context of higher education.“*

 In my experience, and assuming person centred care through communication is the outcome, curriculum/game designers need to give learners increasing doses of reality to learn/practice communicating in a person centred manner. A schema of progression might be - 

 Paper play -> Virtual play -> Simulation -> Real world context (simple) -> Real world (complex).

*Respond: We have clarified the progression in the game through virtual play to real world.* *“the player can choose challenges within the game or in real world. When the player is within the game, he/she encounters virtual patients in a home environment (level 2), in a hospital context (level 3), in a primary care context (level 5), and in a digital meeting context (level 7). The meetings with virtual patients aim to train the player’s skills in listening, partnership, and establishing a health plan. The tasks encountered in the real world aim to train the player to develop listening and partnership skills in clinical activities (level 4), to identify what tasks can be person-centred at their workplace (level 6), and to establish health plans in clinical contexts (level 8).”*

 So while the virtual context is safe and one can be creative about situations of/for learning, it remains a preliminary, and usually an early step in the train of learning/practice before being entrusted as a professional. The caveat being that simulations allow for the curation and creation of unusual/rare situations useful for learning, particularly useful when the opportunistic context is a rarity eg. rare conditions.

*Respond: In the case we describe, the PCC game was used in a second cycle course which imply that all students had experiences from clinical practice either from clinical placement (internship) or employment. This means that the link to reflection and testing in real world clinical practice are embedded in the course guide for the game (appendix 5).* *The setting has been more clearly described in the Use cases section:* *“All students had experiences from clinical practice either from clinical placement (internship) or employment, which was used in their reflections.”*

 What would strengthen the manuscript would be to show the effects of playing the game on a specific group of learners. I imagine using the virtual context may be more effective for early learners eg. "preclinical students" rather than mature clinical students who have actual interactions with patients, family members, and care teams. And that for the more senior learner who has the opportunity to, reflection on actual experiences may be more directly effective in being critical about their/our (unconscious, reflex) behaviours in the actual work and practice.

*Respond: We agree that this would strengthen the paper but for now this is beyond the scope of this method paper. However, interesting for further research, which has now been added in the end of the discussion.* *“However, the effects on learning PCC by playing the game needs to be further evaluated.”*  

 In conclusion, my sense is that this serious game shows promise possibly for the early learner; and while I am less convinced of its value for the more senior care professional, this is merely my opinion. The answer will reveal itself in a carefully planned pilot with clear outcome goals/measures so that the effects of the PCC game in the serious work of person centred care will be made clearer for health professional educators.

*Respond: Thank you for your suggestions and valuable advises which we will keep in mind for future evaluation of the PCC game.*

# Peer review: Reviewer response for version 1

**DOI:** 10.21956/mep.20753.r33279

**Published:** 2023-06-15

**Authors:** Margaretha Ekebergh

**Affiliations:** 1 Faculty of Caring Science, Work Life and Social Welfare, University of Borås, Borås, Sweden

Thank you for asking me to review this article. This is an innovative and interesting article. It is a new approach of learning through reflection. I think this can have benefits of students’ learning, but I have some considerations regarding the reflection. I miss a clear definition of the concept of reflection. What is reflection actually? It is very important to have this knowledge to handle and understand the different levels of the method. From my point of view, reflection is not the same as “discussion” and it is not the same as “questions with answers”. If you do not have this very clear the method with the games might be understood as a problem solving method, and what I have understood is this not correct. The authors use Dewey as a theoretical base, but I think the concept could be more explained.

 According to above, the content of the article should be strengthened through an explicit explanation of how the games support the reflection in the learning process. What is the difference in relation to use patients’ narratives for reflection? Is it merely a fun activity to use games, or can the students expect a better support for the learning, then using patient narratives in an ordinary way? Where is the "group reflection", which research shows has a very good impact on learning?

 These comments could be helpful to strengthen the mission with the care games. However overall, I find the article well done and of course, I want to encourage new development of didactics to support reflection.

Is the rationale for developing the new method (or application) clearly explained?

Yes

Is the description of the method technically sound?

Yes

Are the conclusions about the method and its performance adequately supported by the findings presented in the article?

Partly

If any results are presented, are all the source data underlying the results available to ensure full reproducibility?

Yes

Are sufficient details provided to allow replication of the method development and its use by others?

Partly

Reviewer Expertise:

Reflection and learning in caring contexts. Lifeworld oriented caring science with focus on the patient perspective.

I confirm that I have read this submission and believe that I have an appropriate level of expertise to confirm that it is of an acceptable scientific standard, however I have significant reservations, as outlined above.

# Response

**Published:** 2023-07-11

**Authors:** Caroline Feldthusen

**Affiliations:** University of Gothenburg, Sweden

*The authors would like to thank the reviewer for valuable comments and suggestions which will enhance the manuscript and make the article clearer.*Point by point respond: 

*Our answer on your comment “what is refection actually”* *Thank you for this comment. We have now explained the concept reflection as described by Dewey. We agree with you that reflection is not a “discussion” or a “question with answers”. So, we suggest adding the following definition to reflection to the text.* *“Dewey argues that reflection is a process that entails individuals actively, persistently, and carefully examining, questioning, considering, and evaluation their thoughts and ideas in order to arrive at conclusions that can offer more profound understanding, i.e., to have grasped the meaning of something. In this way, reflection guides future action and thinking.”*

*Our answer on your comments about “strengthen how games support student reflection”, “what is the difference between using refection in games and patients narratives” and about the effects of group reflection.* *We agree that we can strengthen the mission of the PCC game by adding how games support reflection, the difference to use reflection in games or narratives and add text about group reflection.* *Our suggestion to change in the manuscript:* ***Serious game*** *Various learning activities have been developed to implement PCC (Britten et al. 2020). One of these activities is called the PCC game. Games that are used to change the players' thinking or skills are called ‘serious games’. The overall purpose of serious games is to promote learning in a pleasurable way through reflection (Laamarti et al. 2014). Games have been used in higher education (Sharifzadeh et al. 2020) to increase student academic commitment, retention, performance (Pechenkina et al. 2017), thinking, emotions (Vlachopoulos and Makri 2017), as well as motivation and academic skills (Subhash and Cudney 2018; Zeybek and Saygi, 2023). Further, a systematic review by Krishnamurthy, has shown that games enhance students' individual and group-based reflective abilities (Krishnamurthy et al., 2022), indicating that serious games can support students` reflection*

# Peer review: Reviewer response for version 1

**DOI:** 10.21956/mep.20753.r32911

**Published:** 2023-02-15

**Authors:** Clare Killingback

**Affiliations:** 1 Faculty of Health Sciences, Department for Sport, Health and Exercise Science, University of Hull, Hull, UK

Thank you for asking me to review this very interesting method article. It presents an innovate pedagogic approach to teaching person-centred care whilst drawing strongly on reflective practice skills. I love the idea of using gamification to teach person-centred practice and imagine it must have taken considerable time and effort to progress to this stage with the broad range of stakeholders involved so I wish to congratulate the team on this endeavor - well done!

 I offer a few comments here with the intention of strengthening the manuscript.

 1. I was initially a bit confused by the abstract as to whether the focus of the method was on developing reflective practice or on person-centred care skills (both quite complex constructs). Having read the full article and extended data it appears to be more about teaching PCC by using reflective skills (if I've understood correctly). Please could more detail be added to the abstract to clarify this and throughout the article to ensure that the focus is on PCC as supported by reflection.

 2. It looks like the game has been developed based on the Gothenburg framework of PCC. Please can a summary of this framework be included here to add context to the study. This helps me understand whether the game will be transferrable to other contexts / frameworks.

 3. I was confused that the game was "designed for professionals, patients and relatives of patients." I thought the focus was on health care students? The level 1 questions certainly seem to focus on those who work in healthcare rather than patients or relatives. Please could this be clarified in the text?

 4. How has this been tested with students? I appreciate this is a methods paper but some details on the proposed pedagogic evaluation would add confidence of the plan to test the game further for supporting a change in practice for students.

 5. I see in the extended data it keeps the types of healthcare professionals this is aimed at deliberately broad. Again, a bit more detail on who was involved in the design, i.e. which healthcare students were involved (I assume it would have been a broad range if the goal was for a broad audience) helps me as a reader judge the transferability to my student population.

 6. As an educator I'd be keen to hear how it has been embedded into the curriculum. Which level of study is it aimed at, is there a summative assessment component (I know from my own students that assessment drives learning!), or is it purely formative. Which links to my next point...

 7. Any initial summary stats on level of engagement from students? How many who were expected to engage did download it and engage, how many progressed through all the levels etc. Again, I know this is a methods paper so may be beyond the scope but I imagine this would have been tested so commenting on this would be helpful.

 I do really love the idea of this so thank you for sharing your innovative and creative ways to teach PCC. I did a review myself last year of all the ways that I found in the literature to teach PCC in physiotherapy and the literature was quite limited so I welcome this addition.

 Kindest regards,

 Clare

Is the rationale for developing the new method (or application) clearly explained?

Yes

Is the description of the method technically sound?

Yes

Are the conclusions about the method and its performance adequately supported by the findings presented in the article?

Partly

If any results are presented, are all the source data underlying the results available to ensure full reproducibility?

No source data required

Are sufficient details provided to allow replication of the method development and its use by others?

Yes

Reviewer Expertise:

Person-centred care, pedagogic research, physiotherapy

I confirm that I have read this submission and believe that I have an appropriate level of expertise to confirm that it is of an acceptable scientific standard, however I have significant reservations, as outlined above.

# Response

**Published:** 2023-07-11

**Authors:** Caroline Feldthusen

**Affiliations:** University of Gothenburg, Sweden

*The authors would like to thank the reviewer for valuable comments and suggestions which will enhance the manuscript and make the article clearer.* Point by point respond: 1. I was initially a bit confused by the abstract as to whether the focus of the method was on developing reflective practice or on person-centred care skills (both quite complex constructs). Having read the full article and extended data it appears to be more about teaching PCC by using reflective skills (if I've understood correctly). Please could more detail be added to the abstract to clarify this and throughout the article to ensure that the focus is on PCC as supported by reflection.

*Respond: We agree, this is not clear. The focus is on learning PCC through reflection. We have revised the abstract to clarify this.* *In the abstract we have added:* *- “Person-centred care (PCC) is being implemented within many health care systems and educational institutions are important enablers of learning PCC.”* *- the PCC game was designed to promote “learning of PCC by reflection”,* *- and finally, that the PCC game could be used to induce “PCC knowledge and skills by” student reflection in an academic course on PCC.* *We hope this will make the focus of the article clearer.*

 2. It looks like the game has been developed based on the Gothenburg framework of PCC. Please can a summary of this framework be included here to add context to the study. This helps me understand whether the game will be transferrable to other contexts / frameworks.  

*Respond: Yes, this is true. The GPCC framework and the cornerstones listening to patient narratives, partnership and documentation of a health plan is the basis for the PCC game. The GPCC framework has now been outlined in the method section, development of the PCC game.* *Added in the manuscript:* *“The theoretical framework of the PCC game is grounded in the work of the Gothenburg centre for person centred care (GPCC) which is built upon Ricoeur's ethic (Ekman et al., 2011). This ethic declares that every person has the right to use their capacity, express their will and decide for themselves what constitutes a good life. Further, the GPCC framework* *(Ekman et al., 2011; Brittan et al., 2020)* *suggests that the ethics may be practiced by the three cornerstones listening to patient narrative, partnership, and documentation of a health plan. The core component of PCC is based on mutual respect and the co-creation of care through a partnership between patients, their families and careers, and health care professionals (Ekman et al., 2011). “*

 3. I was confused that the game was "designed for professionals, patients and relatives of patients." I thought the focus was on health care students? The level 1 questions certainly seem to focus on those who work in healthcare rather than patients or relatives. Please could this be clarified in the text?  

*Respond: The PCC game was developed for professionals, patients, and relatives to promote learning and reflection about PCC. In the game you choose version for either healthcare professionals or lay person. We wanted to try the version for health professionals in a higher education learning context and have therefor used it in health care students in a course on advanced level to promote their reflection and learning PCC. This is the case we describe in this method paper. We have tried to clarify this in the method section.* *Added in the manuscript:* *“The PCC game was designed primarily for professionals, but also for patients and relatives and you choose to enter the game either as a healthcare professional or a lay person. The healthcare professional entry can also be used to promote healthcare students reflection and learning about PCC in the context of higher education.”*

 4. How has this been tested with students? I appreciate this is a methods paper but some details on the proposed pedagogic evaluation would add confidence of the plan to test the game further for supporting a change in practice for students.

*Respond: "Students from the Swedish University of Gothenburg, at medicine and nursing participated both in the development of the game and testing the prototype and final version by written comments."* *This has been added to the method section, development of the PCC game.* *Further, the PCC game was tested in an interdisciplinary, higher education course including students from nursing, medicine, occupational therapy, physical therapy, pharmacy, dietetics, and speech therapy. This has been clarified in the use cases section.* *In the manuscript:* *“We tested the PCC game in an interdisciplinary, higher education course including students in the process of becoming nurses, physicians, occupational therapists, physical therapists, pharmacists, dietitians, and speech therapists. All students had experiences from clinical practice either from clinical placement (internship) or employment, which was used in their reflections.*

*One example for the second cycle course; Person-centredness in health and social care, is shown in Appendix 4. We conducted the course in five modules, which were accompanied by a standardized course guide (Appendix 5). All students played all nine levels of the game over a period of five weeks (Table 1). In each module, the students were given tasks that increased the degree of reflection. Formative assignments and examinations regarding the students’ progression through the game were used.”*

   5. I see in the extended data it keeps the types of healthcare professionals this is aimed at deliberately broad. Again, a bit more detail on who was involved in the design, i.e. which healthcare students were involved (I assume it would have been a broad range if the goal was for a broad audience) helps me as a reader judge the transferability to my student population.

*Respond: Healthcare students from nursing and medicine participated in the development and testing of the game which has now been added in the method section. However, the game was tested in an interdisciplinary course as described above.*

 6. As an educator I'd be keen to hear how it has been embedded into the curriculum. Which level of study is it aimed at, is there a summative assessment component (I know from my own students that assessment drives learning!), or is it purely formative. Which links to my next point...

*Respond: The PCC game has not been embedded in undergraduate national study curriculum or local program syllabuses for healthcare professions. However, it was included in a local course syllabus offered as an interdisciplinary contract education in second cycle health education programs. In this course formative assignments and examinations regarding the students’ progression through the game were used.* *In the manuscript:* *“All s* *tudents played all nine levels of the game over a period of five weeks (Table 1). In each module, the students were given tasks that increased the degree of reflection. Formative assignments and examinations regarding the students’ progression through the game were used.”*

 7. Any initial summary stats on level of engagement from students? How many who were expected to engage did download it and engage, how many progressed through all the levels etc. Again, I know this is a methods paper so may be beyond the scope but I imagine this would have been tested so commenting on this would be helpful.

*Respond: All students downloaded the app and reported progression by submitting their reflections and assignments in the game, this was part of the examination. This has been clarified in the use cases section.* *In the manuscript:* *“All s* *tudents played all nine levels of the game over a period of five weeks (Table 1). In each module, the students were given tasks that increased the degree of reflection. Formative assignments and examinations regarding the students’ progression through the game were used.”*
